# Delta and Theta Band Power Alterations During Face and Face Pareidolia Perception in Children with Autism Spectrum Disorder: An Electroencephalographic Analysis

**DOI:** 10.3390/medicina61040754

**Published:** 2025-04-19

**Authors:** Gülsüm Akdeniz

**Affiliations:** 1 Department of Biophysics, Faculty of Medicine, Ankara Yıldırım Beyazit University, Ankara 06800, Türkiye; gulsumakdeniz@aybu.edu.tr; 2 Department of Neuroscience, Institute of Health Science, Ankara Yıldırım Beyazit University, Ankara 06800, Türkiye

**Keywords:** autism spectrum disorder, face processing, pareidolia, EEG, N170

## Abstract

*Background and Objectives*: Autism Spectrum Disorder (ASD) is characterized by a range of deficits across cognitive, sensory, motor, emotional, language, and social domains, which can significantly hinder daily functioning and social interactions. This study explores the differences in brain activity between children with ASD and typically developing peers, focusing on their responses to face and face pareidolia stimuli. *Materials and Methods*: A group comprising ten typically developing children (four males, six females), aged between 6 and 16 years, alongside eleven children diagnosed with ASD (three males, eight females), whose ages ranged from 6 to 15 years, were engaged in the pilot study. We recorded brain electrical activity using electroencephalography (EEG) while participants viewed images of face and face pareidolia images. Following face and pareidolia stimulus presentation, delta and theta powers in the 0.5–4 Hz and 4–6 Hz frequency ranges and within the 140–190 ms time window were analyzed for both typically developing children and children with ASD. *Results*: The research result reveals that children with ASD show lower amplitude and delayed latency in their EEG responses, particularly in the theta and delta frequency bands, when processing images that evoke face pareidolia. *Conclusions*: The findings suggest that while children with ASD face challenges in recognizing faces, they may still possess some perceptual abilities that could be harnessed for therapeutic interventions. Moreover, this research highlights the potential of the face pareidolia paradigm to provide insights that could inform future strategies aimed at enhancing social attention and interaction skills in children with ASD. Despite the limitations of the current sample size, this study provides a valuable foundation for future investigations. Expanding the participant pool will be crucial for confirming and generalizing these findings.

## 1. Introduction

Autism Spectrum Disorder (ASD) is a neuropsychiatric condition characterized by congenital impairments or deviations in social interaction, communication, and cognitive development, typically emerging in the early years of life. Children with ASD often exhibit deficiencies in social communication skills, reluctance to engage in interactions, and avoidance of eye contact [1]. ASD is characterized by deficits in the cognitive, sensory, motor, emotional, language, and social domains. Individuals with ASD often exhibit lower cognitive and academic performance, particularly in literacy, mathematics, and comprehension skills, along with difficulties in daily functioning, self-expression, and understanding others’ emotions [2,3,4]. Approximately 50% of individuals with ASD display varying degrees of intellectual impairment, with attention deficits, hyperactivity, and communication difficulties impacting standardized intelligence test performance, necessitating alternative observational assessments [5,6]. Sensory processing abnormalities include reduced responsiveness to environmental stimuli, atypical visual attention patterns, hypersensitivity such as discomfort from cold water, or hyposensitivity such as unresponsiveness to pain [7,8,9,10,11]. Motor deficits affecting fine and gross motor skills can significantly restrict daily activities, with impairments in balance, coordination, spatial awareness, and repetitive motor behaviors, such as toe walking or hand-flapping [8,9,10,11]. Early interventions, including physical and occupational therapy, are crucial for improving motor coordination and problem-solving skills [9,12]. Emotional regulation difficulties, particularly in expressing happiness, negatively impact social interactions and parent–child relationships [13,14]. Language and communication impairments, such as limited eye contact, joint attention deficits, and restricted use of gestures, hinder effective interaction and expression of needs [15,16,17,18,19]. Social skill deficits, including difficulties in imagination, visual perception, and imitation, lead to challenges in peer relationships, social isolation, and behavioral issues, further affecting academic and daily life participation [20,21]. These multifaceted impairments emphasize the need for early diagnosis and tailored interventions to support the cognitive, motor, social, and emotional development of individuals with ASD.

The human face serves as a fundamental focal point for visual attention and perception from birth. Newborns demonstrate innate sensitivity to faces and face-like stimuli, suggesting the presence of a subcortical neural system that directs visual attention to faces from the earliest stages of life. Additionally, the physical characteristics of the face, particularly the eyes, naturally attract attention [22]. One of the early and prominent indicators of ASD is reduced eye contact during social communication and interactions, making it a critical marker for early diagnosis. The absence of eye contact, a core symptom of ASD, is believed to be linked to the underlying mechanisms of symptom development [23]. While typically developing individuals establish eye contact as a crucial aspect of effective communication, research indicates that individuals with ASD engage in significantly less eye contact during interactions [24]. Furthermore, studies suggest that children with ASD exhibit slower processing and reduced ability to detect and direct gaze compared to their typically developing peers [25]. However, one study contradicts these findings, suggesting that gaze-related impairments may not be universally present in ASD [26]. Additionally, another study proposed that difficulties in processing the structural features of faces contribute to deficits in face recognition and learning in individuals with ASD [27]. Individuals with ASD exhibit reduced activation of the fusiform face area (FFA) during face recognition tasks [28]. Typically, right FFA activation is associated with face recognition, and lesions in this region result in impaired face perception. Additionally, significant differences in right fusiform gyrus volume have been identified in individuals with ASD, further highlighting the neuroanatomical variations related to face processing difficulties [29].

Pareidolia is defined as a common tendency to perceive meaningful patterns in ambiguous or low-clarity images, such as clouds, landscapes, shadows, or inkblots, even when no actual pattern exists [30,31]. Face pareidolia, specifically, refers to the phenomenon in which the brain perceives a non-face stimulus as a face, despite the absence of an actual face structure [32]. The relationship between face pareidolia and ASD reveals significant differences in face perception abilities. A study has shown that children with ASD exhibit slower reaction times and lower accuracy in identifying both real and pareidolic faces than typically developing peers [33]. Additionally, they identify significantly fewer pareidolic faces, suggesting a specific deficit in attending to faces [34]. Neurophysiological findings indicate that transcranial alternating current stimulation study, which enhances gamma band oscillations, can improve face pareidolia recognition in healthy individuals, emphasizing the role of neural mechanisms in face perception [35]. Moreover, individuals with autism exhibit perceptual integration deficits, which may hinder their ability to process faces as meaningful wholes, ultimately affecting their social interactions [36]. However, despite these deficits, research suggests that once directed towards face-like objects, children with ASD can perceive them holistically, indicating some preserved perceptual capacities [37]. Considering the existing research findings on children with ASD, there remains a need for a quantitative method to accurately assess face perception in this population.

Event-related potentials (ERPs) are widely used neurophysiological measures to study perceptual and cognitive processes related to face perception and recognition. The N170 component, a negative ERP typically peaking around 170 ms post-stimulus onset, is a key focus in face processing research [38]. Characterized by its greater amplitude and shorter latency in response to human faces compared to other visual categories [39], the N170 is considered a reliable index of face-specific neural processing [40] and is thought to reflect the structural encoding of face information [38,41]. Furthermore, the N170 offers the advantage of precise temporal tracking of neural processes engaged during face perception, providing critical insights into the early stages of visual information processing related to faces [38]. By capturing the timing of these neural events, researchers can delineate the initial stages of face encoding. Wavelet analysis is a technique of time–frequency analysis that decomposes a signal into its constituent frequencies and examines the variation in these frequencies over time. The integration of ERP methodology with wavelet analysis offers a synergistic approach, potentially yielding a more comprehensive understanding of the intricate neural mechanisms that underlie both the perception and subsequent recognition of faces. This combined approach allows for the examination of both the time-locked neural responses (ERPs) and the underlying oscillatory dynamics (wavelet analysis), providing a richer characterization of the neural activity associated with face processing. In summary, the N170 component serves as a valuable neurophysiological marker for studying face processing, offering insights into both typical and atypical development of face perception abilities [41,42]. Its sensitivity to various factors and reliability across studies make it an essential tool in face perception research. Our research questions required an analytical approach capable of addressing both the temporal and frequency characteristics of EEG signals associated with face pareidolia. We selected EEG wavelet analysis due to its superior temporal resolution compared to traditional spectral methods, enabling us to accurately determine the timing of specific frequency band activities in relation to the experimental paradigm.

We aim to identify differences in visual perception in children with ASD by investigating the electrical brain activity in response to face pareidolia stimuli compared to their typically developing peers.

## 2. Materials and Methods

### 2.1. Participants

Children in the ASD group were assessed and diagnosed by a qualified clinician utilizing the criteria established in the Diagnostic and Statistical Manual of Mental Disorders, Fifth Edition (DSM-5) [1]. Both children with ASD and typically developing children were devoid of any comorbid disorders or diseases. Furthermore, comprehensive physical and neurological evaluations conducted on all participants yielded normal results, and none of the subjects were under pharmacological treatment. Table 1 presents the demographic data of the children involved in the study. The mean performance IQ scores of both children groups demonstrate similarity. Attention deficit symptoms were observed to be more pronounced in children with autism than in their neurotypical counterparts, with parents reporting incidences of maladaptive behaviors exhibited by their children. Participants in both groups had no additional disabilities such as visual or hearing impairments, were able to follow instructions, and could complete the given task. Exclusion criteria included being outside the 6–17 years age range, unwillingness to participate, inability to follow instructions, presence of claustrophobia, or having a chronic illness.

After providing detailed information about the study, written and verbal informed consent was obtained from the participants and their caregivers, and electroencephalography (EEG) recordings were conducted in a Faraday cage at the Neuroscience Laboratory of the Ankara Yıldırım Beyazıt University Faculty of Medicine, using the face pareidolia paradigm. Ethical approval was obtained from the Clinical Research Ethics Committee of SBÜ Ankara Dr. Sami Ulus Maternity and Children’s Health and Diseases Training and Research Hospital (Protocol No: E-21/02-93).

### 2.2. EEG Recording

EEG recordings were conducted in an electrically shielded Faraday cage at the Neuroscience Laboratory of Ankara Yıldırım Beyazıt University Faculty of Medicine. The Brain Products ActiCHamp EEG system was used with 32-channel Ag-AgCl electrodes, placed according to the international 10–20 system. Reference electrodes were positioned at the earlobes and Cz, while electrooculography electrodes were placed at the outer canthus and supraorbital area of the right eye to monitor eye movements. Impedance was maintained below 5 kΩ, and bandpass filtering (0.5–100 Hz) was applied, with a sampling rate of 1 kHz.

### 2.3. Stimuli and Experimental Design

**Figure 1:**
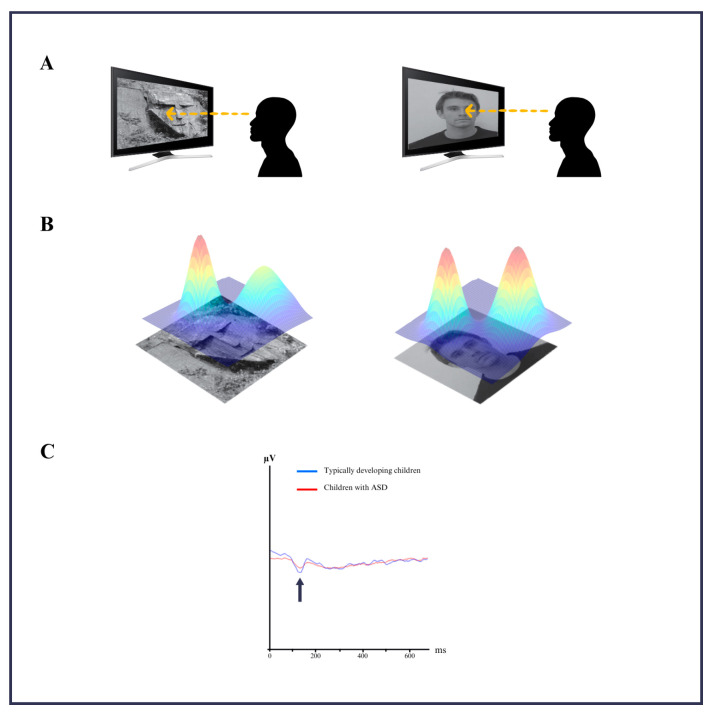
(**A**) Representation of the face and face pareidolia images used in the experimental design. (**B**) A schematic representation of the 3D density plots of face and face pareidolia images, as used during EEG recording. (**C**) N170 responses related to pareidolia in the typically developing children compared to the children with ASD by data recorded from electrodes P8, P7, TP7, and TP8.

The experimental design included 40 randomly presented images of faces and face pareidolia images that were displayed for 1000 milliseconds, with an interstimulus fixation period of 1000 milliseconds (Figure 1A). Participants were instructed to press a button when they perceived a face or a face pareidolia image. To minimize eye movement artifacts, a red fixation point on a black background was displayed between stimuli. Each EEG session lasted approximately 2 min and 40 s per block.

### 2.4. EEG Data Analysis

The 3D density plots of face or face pareidolia images used in the EEG recording are shown in the schematic diagram in Figure 1B. EEG data were segmented into epochs from -400 to +1000 ms relative to the stimulus onset. The preprocessing included artifact rejection, bandpass filtering (0.5–30 Hz), and notch filtering (50 Hz). Event-related potential (ERP) analysis focused on the N170 component to assess cortical responses. Additionally, wavelet transform analysis was employed to examine the time–frequency dynamics with improved temporal resolution.

### 2.5. Statistical Analysis

Comparing the ASD and TD groups for the face and pareidolia condition, independent-samples *t*-tests were performed to evaluate group differences in ERP amplitudes (N170) and time–frequency power (delta and theta bands). For ERP data, mean amplitudes were extracted from the 140–190 ms window over the P8, P7, TP7, and TP8 electrodes. For time–frequency analyses, mean power values in the delta (0.5–4 Hz) and theta (4–7 Hz) bands were calculated over relevant time intervals. All statistical analyses were performed using Statistical Package for the Social Science (Version 27), and results were considered significant at *p* < 0.05.

## 3. Results

**Figure 2:**
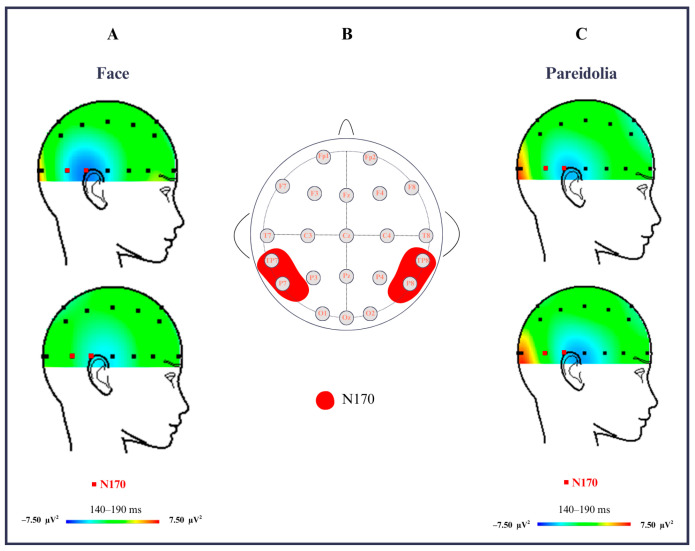
(**A**) Scalp distribution of the N170 component (140–190 ms) in response to face stimuli for ASD group. Red squares indicate regions of significant N170 activation. (**B**) Electrode locations used for N170 analysis (P7, P8, TP7, TP8), highlighted in red. (**C**) Scalp distribution of the N170 component in response to face stimuli for ASD group. Red squares indicate regions of significant N170 activation.

The ASD group comprised six males and four females (mean age = 7.1), while the typically developing children included eight males and three females (mean age = 6.9). All participants had no comorbid illnesses, no claustrophobia, and were right dominant handed. Mean reaction time and accuracy rates for both groups are presented in Table 1. The ASD group exhibited lower amplitude and delayed latency compared to the control group when analyzing pareidolia-related EEG responses. This was observed in the data recorded from electrodes P8, P7, TP7, and TP8 in the N170 brain-wave component (Figure 1C). The scalp distribution of activity during face stimuli is shown in Figure 2A, and pareidolia stimuli in Figure 2C. The electrode positions and region of interest for N170 analysis are highlighted in Figure 2B.

### 3.1. Time and Frequency Analysis Results

Time–frequency analysis of event-related potentials (ERPs) in the delta and theta frequency bands revealed significant differences in total and evoked power. A *t*-test was performed for statistical analysis to compare four conditions: children with ASD and typically developing children for face stimulus, children with ASD versus typically developing children for face pareidolia stimulus, children with ASD for face stimulus versus face pareidolia stimulus, and typically developing children for face stimulus versus face pareidolia stimulus. Significant differences were identified (*p* < 0.05).

#### 3.1.1. Delta Power

**Figure 3:**
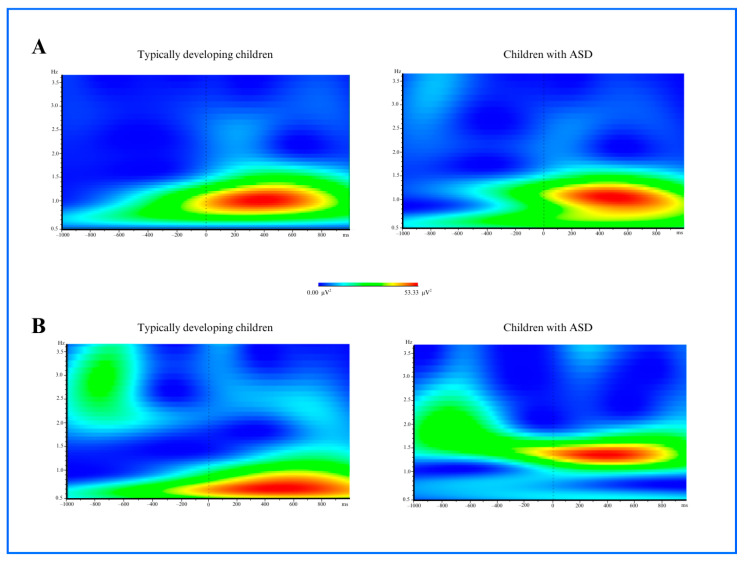
(**A**) Delta power analysis images of 2–4 Hz frequency and in 140–190 ms time regarding typically developing children and children with ASD group after the face stimulus (*p* = 0.011). (**B**) Delta power analysis images of 2–4 Hz frequency and in 140–190 ms time regarding typically developing children and children with ASD group after the face pareidolia stimulus (*p* = 0.009).

For both face and pareidolia stimuli responses, a significant difference in delta power (2–4 Hz frequency, 140–190 ms time window) was observed in the frontal region between the ASD and control groups when comparing the average activity recorded from electrodes Fz, FC3, and FC4 (face stimuli: *p* = 0.011, pareidolia: *p* = 0.009). In response to face stimuli, TD children showed increased delta power beginning at approximately 100 ms post-stimulus, with the effect persisting until around 400 ms. In children with ASD, a similar onset was observed, while the delta response was prolonged, lasting up to 550 ms. For pareidolia stimuli, TD children exhibited delta activity starting around 100 ms, which declined by 400 ms, whereas children with ASD showed a delayed onset around 150 ms and a more sustained response extending until 600 ms. Statistical analysis revealed that the ASD group exhibited significantly lower amplitude compared to typically developing individuals (face stimuli: *p* = 0.011, pareidolia: *p* = 0.009). These findings are illustrated in Figure 3.

#### 3.1.2. Theta Power

**Figure 4:**
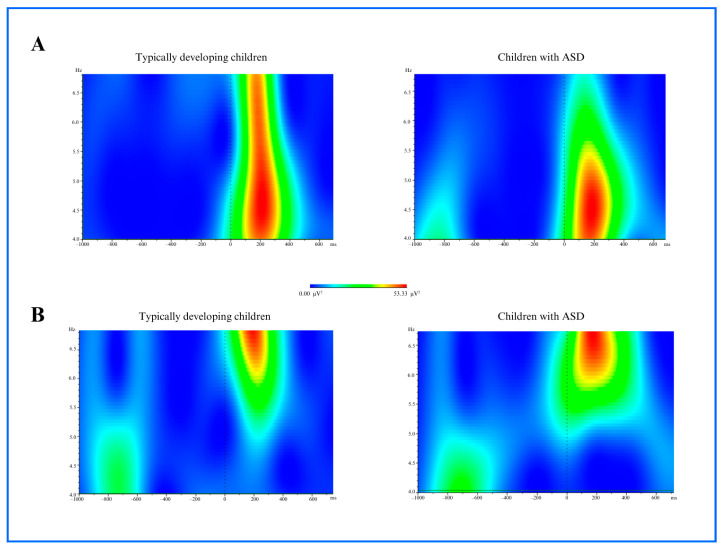
(**A**) Theta power analysis at 4–6 Hz frequency within the 140–190 ms time window for typically developing children and children with ASD group after the face stimulus (*p* = 0.019). (**B**) Theta power analysis at 4–6 Hz frequency within the 140–190 ms time window for typically developing children and children with ASD group after the face pareidolia stimulus (*p* = 0.007).

A significant difference in theta power was observed in the frontal brain region, as measured from the Fz, FC3, and FC4 electrodes, when comparing the control and ASD groups (face stimulus: *p* = 0.019, pareidolia: *p* = 0.007). Theta activity in TD children during face stimulus processing emerged at approximately 100 ms and subsided by 300 ms. In contrast, children with ASD exhibited a delayed theta response around 150 ms onset, which lasted up to 450 ms. For pareidolia stimuli, both groups showed theta activation beginning around 150 ms. While TD children’s theta power diminished by 350 ms, children with ASD maintained enhanced theta activity until approximately 500 ms. The ASD group exhibited a decreased amplitude and delayed latency compared to typically developing individuals. The theta power distribution, particularly prominent in the frontal region, is illustrated in Figure 4.

## 4. Discussion

Our research is the first quantitative analysis of the brain dynamics of face and face pareidolia perception, comparing children with ASD to typically developing children, using EEG methodology. The results of this study indicate that the relationship between face pareidolia and ASD is particularly noteworthy. While children with ASD exhibited slower reaction times and decreased alterations in power spectra in brain dynamics compared to typically developing children, their ability to perceive face-like objects holistically suggests some preserved perceptual capacities. Our striking finding underscores the importance of early diagnosis and tailored interventions for children with ASD, focusing on enhancing cognitive, motor, social, and emotional development. We suggest that while there are significant deficits in face processing, there may be underlying neural mechanisms that could be used for therapeutic interventions aimed at improving social attention and interaction skills in children with ASD. The unique insights gained from the face pareidolia paradigm may inform future research and intervention strategies aimed at supporting social and communicative development in children with ASD.

The processes that integrate information from the senses to create a face perception, the integrative top-down processing process involving long connections, have been reported to be associated with delta and theta waves [43,44]. Our findings indicate that the difficulties experienced by individuals with ASD in top-down processing may affect their difficulty in perceiving faces. Comparisons between the ASD group and their typically developing peers revealed significant differences in delta and theta powers in response to face images. This may be due to individuals with ASD not looking at real faces. According to our results, the fact that the ASD group did not look at real faces was interpreted as their misperception of the social message or their intentional unwillingness to look. A study investigating ERP during face processing in individuals with ASD has consistently demonstrated a delayed and broadened N170 response in the occipitotemporal cortex, coupled with a diminished sensitivity to inverted faces, reflecting a distinct N170 waveform compared to typically developing children [45]. Another study found that individuals with ASD fixate on different parts of the face compared to their typically developing peers, which may lead to differences in evoked N170 amplitudes and latency [46]. This difference in N170 amplitude and latency is thought to be due to a lack of cortical connectivity in working memory related to face processing in children with ASD [45,47]. Working memory is known to be associated with the middle frontal gyrus, inferior frontal gyrus, and inferior parietal regions. Activation has been observed in these regions and the FFA in both individuals with ASD and typically developing individuals. However, activation in the frontal regions was predominantly limited to the right hemisphere in individuals with ASD, whereas frontal activation in the control group was bilateral.

Studies using face pareidolia images allow us to investigate the brain conditions of individuals with face perception problems. The use of the face pareidolia paradigm in children with ASD prevents the emergence of factors that might trigger face expressions or physiological responses typically associated with real faces [37]. Our study revealed that children with ASD exhibited the lower amplitude and delayed latency of the N170 wave in response to pareidolia stimuli compared with typically developing children, which suggests that children with ASD are less likely to direct their attention towards face pareidolia images. Research indicates that children with ASD tend to avoid focusing on faces, unlike their typically developing peers [48,49]. This lack of attention is associated with a deficiency in face processing, which contributes to their challenges in recognizing and perceiving faces [34]. Our findings underline that the lower amplitude and delayed latency of the N170 wave in response to pareidolia stimuli in children with ASD reveal that children with ASD tend to avoid focusing on not only faces but also face-like objects such as face pareidolia, contributing to their challenges in recognizing and perceiving faces. The delayed latency of the N170 response to face pareidolia indicates that although pareidolias are perceived as faces, the process is slower. This study has shown that face stimuli activate the superior temporal sulcus, FFA, and lateral temporal regions in the brain. Additionally, N170 responses were observed for both faces and face pareidolia. Integrating our findings with the existing literature, we conclude that the tendency of individuals with ASD to avoid looking at faces or not to respond to faces limits their communication. They have difficulty interacting with individuals around them. This situation is thought to cause social isolation in children with ASD.

A study involving the presentation of pareidolic faces on a screen reported that children with ASD exhibited reduced sensitivity to face pareidolia images compared to typically developing children [34]. Simultaneously, it was stated that while children with ASD gradually improved in their ability to recognize faces in pareidolia images as they grew up, their performance was still lower than that of typically developing children [34]. Furthermore, another study stated that children with ASD are unable to orient their attention towards or recognize faces in pareidolia images appearing in landscapes, nature, or sky [34]. The findings of Ryan et al.’s study support our results. Our time–frequency analysis of delta and theta frequency bands revealed significant differences in delta and theta power between the children with ASD and typically developing children in response to face pareidolia images. Specifically, children in the ASD group showed significantly lower delta and theta powers in the frontal regions, which are associated with cognitive control and emotional regulation [50]. This is particularly relevant considering that emotional regulation difficulties are common in children with ASD, affecting their social interactions and relationships [51]. The prolonged latency and reduced amplitude in the delta and theta frequency bands observed in our results suggest a delay in cognitive processing and emotional responses, which could contribute to understanding the social communication challenges faced by children with ASD.

In our study, we identified a notable reduction in delta power within the frontal region of children with ASD compared to typically developing children, in response to both face and face pareidolia stimuli. This observation is consistent with the existing literature that suggests atypical low-frequency oscillatory activity in individuals with ASD during social cognitive tasks [52]. Delta oscillations are associated with attentional allocation and top-down processing [53] and the diminished delta power observed in the ASD group may indicate differences in the early engagement of these mechanisms when processing socially relevant stimuli, including ambiguous pareidolia images. This may suggest a potentially less pronounced initial orienting response or an alternative mode of attentional deployment towards face information in ASD. Kang et al. [54] found increased connectivity diversity in ASD children within and between brain hemispheres at delta and alpha frequencies, suggesting altered neural network dynamics potentially impacting face processing. Moreover, our analysis of theta power in the frontal region revealed decreased amplitude and delayed latency in children with ASD compared to their typically developing counterparts, again for both face and face pareidolia stimuli. Theta oscillations are commonly linked to cognitive control, working memory, and the processing of salient stimuli [55]. The observed reduction and delay in theta power in the ASD group might reflect differences in the recruitment of these control-related processes during the perception of both real and illusory faces. This could manifest as altered attentional modulation or a less efficient engagement of cognitive resources when evaluating the social significance of facial cues. Notably, while significant differences in delta and theta power were identified, the topographical maps suggested subtle variations in spatial distribution across face and pareidolia stimuli within each group. The differences in low-frequency oscillatory activity may contribute to the altered social perception often associated with ASD, supporting theories that atypical neural synchronization in frontal areas underlies social cognitive challenges [56]. Overall, our findings highlight distinct alterations in delta and theta power during the processing of both real and illusory faces in children with ASD; these differences in early brain dynamics may reflect atypical attentional and cognitive control processes that contribute to the altered social perception characteristic of ASD.

### Limitations

The low number of participants in the study could limit the generalizability of the findings, as a larger sample size would provide more robust data. The implications of these limitations highlight the need for future research to include a more diverse and larger sample size, allowing for a clearer understanding of the differences in EEG responses between children with ASD and their neurotypical peers. Another limitation of the present study is its primary focus on the N170 component of the ERP. While the N170 offers valuable insights into the early stages of face-specific processing, it does not fully capture the broader neural dynamics involved in social cognition. Future research could benefit from examining other relevant ERP components. For instance, the P100, which reflects early visual processing and attention allocation, might reveal group differences in the initial sensory encoding of faces. Furthermore, later components such as the P300, associated with attentional resource allocation and stimulus evaluation, and the late positive potential and vertex positive potential, which occur in a similar time window as the N170 in different brain regions and index sustained processing and emotional appraisal, could provide a more comprehensive understanding of the cognitive and affective processing of faces in children with ASD. Investigating these additional ERP components in future studies may elucidate the temporal evolution of neural differences beyond the initial structural encoding reflected by N170.

## 5. Conclusions

The findings of this study provide significant insights into the differences in electrical brain activity between children with ASD and typically developing peers in response to face pareidolia stimuli. Further exploration of the neural mechanisms underlying these differences could contribute to the development of targeted therapies that leverage preserved perceptual abilities in children with ASD. While children with ASD may struggle with face pareidolia, this phenomenon presents a valuable tool for assessing social attention and perceptual processes in autism, potentially contributing to diagnostic and intervention strategies.

## Figures and Tables

**Table 1:** Demographic details of the participants.

Characteristic	TD Children	Children with ASD
Mean Age (SD)	6.9 (2.0)	7.1 (2.4)
Gender: Female/Male	6/4	8/3
Dominant Hand: R/L	10/0	11/0
Comorbid Illnesses	NA	NA
Claustrophobia	NA	NA
Disruptive Behavior (%)	40.21	23.53
Inattention (M) *	6.05	2.83
Performance IQ (M)	96.42	99.21
Verbal IQ (M)	64.76	97.97
Face Images		
Mean Reaction Time (s)	0.5371	0.5316
Accuracy (%)	68.12	53.33
Face Pareidolia Images		
Mean Reaction Time (s)	0.6930	0.4670
Accuracy (%)	61.25	46.04

SD; Standard Deviation, TD; typically developing, ASD; Autism Spectrum Disorder, M; In months; NA; Not Available * Number of ADHD Inattention Symptoms (DSM-IV-TR); DSM-IV-TR: Diagnostic and Statistical Manual of Mental Disorders (5th ed., text rev.; APA, 2000) [1].

## Data Availability

Data are unavailable due to privacy and ethical restrictions.

## Abbreviations

The following abbreviations are used in this manuscript:

ASD	Autism Spectrum Disorder
EEG	Electroencephalography
ERP	Event-related potential
FFA	Fusiform Face Area
